# Effective Nature-Based Outdoor Play and Learning Environments for below-3 Children: A Literature-Based Summary

**DOI:** 10.3390/ijerph21091247

**Published:** 2024-09-20

**Authors:** Danielle Craig, Nazia Afrin Trina, Muntazar Monsur, Umme Tasnima Haque, Garrett Farrow, Md Zahid Hasan, Fariha Tasnim, Moyin Sabainah Akinbobola

**Affiliations:** Department of Landscape Architecture (DoLA), Davis College of Agricultural Sciences and Natural, Texas Tech University, 2904 15th St., Lubbock, TX 79409, USA; danielle.craig@ttu.edu (D.C.); ntrina@ttu.edu (N.A.T.); uhaque@ttu.edu (U.T.H.); gfarrow@plandcollab.com (G.F.); mdzhasan@ttu.edu (M.Z.H.); fariha.tasnim@ttu.edu (F.T.); makinbob@ttu.edu (M.S.A.)

**Keywords:** infants and toddlers, childcare, nature, outdoor environment, assessment

## Abstract

Early childhood (0–8 years) is a time of rapid brain development supported by spontaneous and informal learning from the surrounding environment. Meaningful contact with nature (a dynamic and varied source of informal learning) during the early years of life sets up rich scopes for such spontaneous learning—especially in the first three years, a period in life that determines all future learning, behavior, and health. Besides its learning affordances, nature-based environments provide numerous health and developmental benefits. Considering that more than 13 million children under 5 years of age in the US spend most of their waking hours in care facilities, the potential benefits of having a nature-based outdoor area in their primary care environments are immense. However, guidelines and assessment standards for designing nature-based outdoor environments for below-three children (infants and toddlers) are almost non-existent. This three-phase research holds the promise of addressing this issue. Phase 1 reviews the available limited literature on below-three children’s outdoor play and learning environments and summarizes their design implications. Phase 2 extracts effective design guidelines and identifies assessment indicators from the Phase 1 studies. In Phase 3, empirical data (environmental assessment data) are collected to compare the existing and proposed design environment conditions of below-3 outdoor play and learning environments in a selected childcare facility. This phase highlights evidence-based assumptions of new criteria, guidelines, and indicators to assess any below-3 nature-based childcare outdoor environments. This research provides new information and insights for designing nature-based outdoor play and learning environments for below-3 children to increase their meaningful connections with natural elements while attending a care facility.

## 1. Introduction

The initial three years of a child’s life are the most critical in terms of their development [1]. Children under three also have unique qualities, particular ways of behaving, and specialized needs that should be considered when designing play and learning spaces [2]. Despite this critical time in child development, relatively little attention has been paid to developing design guidelines for nature-based outdoor play and learning environments (OPLEs) [3]. Creating high-quality nature-based OPLEs for children under three supports the development of their social–emotional, language, cognitive, perceptual, and motor skills [4]. Nature is an essential component of OPLEs for several reasons. It provides universal, unpredictable, bountiful, and beautiful living spaces that connect children to the natural world, stimulate their senses, provide appropriate physical challenges, and inspire their imagination [5,6].

In traditional US childcare facilities, attending children usually spend most of their waking hours indoors. When they go outside, their surrounding environments typically lack the comfort, engagement, and challenges necessary for overall development. Infants and toddlers require OPLEs that provide opportunities for physical development, including crawling, walking, and climbing. They should be free of choking dangers and have a comfortable landing for unsteady feet. Spaces should also allow for safe exploration, peer play, constructive/manipulative play, language learning, and other opportunities associated with their curiosity and cognitive development. Below-3 OLPEs should also accommodate comfort and considerations for accompanying adults, e.g., comfortable seating, shade, natural healing, spaces for relaxation, utility stations, etc. However, many outdoor play places for children are uninteresting, lack natural components, and limit play to a flat, rectangular, rubberized surface [7]. There is limited inclusion of natural landscape elements such as trees, diverse landforms, and vegetation with various shrubs and landcovers, rocks, fallen logs, and loose parts [8,9]. Contrary to popular beliefs that playgrounds only benefit children, dated outdoor playgrounds actually hinder young minds’ potential development due to their confined environment and composition of conventional features [10,11]. Children’s physical, language, and social abilities, as well as creativity, may be enhanced or impaired by their environments [11]. In a nature-based OPLE, nature is incorporated into the learning philosophy, curriculum, and activities, allowing children to engage in immersive experiences in nature [12]. These environments, characterized by their abundant and varied landscape elements, might offer further advancements to children’s health, well-being, and development [12,13]. Unfortunately, written guidance for early care and education programs on designing effective outdoor environments is limited [7].

### 1.1. Developmental Domains for below-3 Children

A high-quality early care setting enables children to engage in active and playful exploration and experimentation [14]. Particularly in the infant and toddler years, the development and learning of children rely on nurturing relationships that are responsive and secure [1]. According to the California Department of Education [15], four major developmental domains during the infant and toddler years are as follows:

- Social–emotional development;
- Language development;
- Cognitive development;
- Perceptual and motor development.

The domains are essential components of early learning and development that contribute to the readiness of young children for schooling (National Research Council and Institute of Medicine, 2000; NAEYC and NAESC/SDE, 2002) [16]. Understanding how varied landscape elements may support each of these domains in a below-3 OPLE is important.

The social–emotional development domain supports the growth of children’s social and emotional capacities, such as interactions and relationships with adults and peers, self-identity, understanding of the child’s own abilities, empathy, impulse control, and emotional expression [15]. For example, in a fruit and vegetable garden, this domain is supported by interactions with adults (i.e., gardening led by a teacher) and among peers (i.e., cooperative gardening) [17]. The language development domain supports children’s understanding of language, how to express themselves, developing verbal and nonverbal communication skills, and engaging with printed language in books and the environment. In a fruit and vegetable garden, this domain is supported by following instructions from the teacher and increasing children’s vocabulary to include food and gardening terms [18]. The cognitive development domain provides a foundation for children to learn cause-and-effect, spatial relationships, problem-solving, classification, and symbolic play, among other things [19]. In a fruit and vegetable garden, this domain is supported by cause-and-effect observation (i.e., if you plant a seed and tend it, then you get food) [20]. The perceptual and motor development domain supports the growth of children’s abilities to become aware of their environments through sensory experiences and to move and coordinate their body using both their large muscles (gross motor functions) and their small muscles (fine motor functions) [21]. In a fruit and vegetable garden, this domain is supported through the children’s involvement in physically planting, tending, observing, and consuming fruits and vegetables [22].

Different design domains provide diverse affordances. According to Gibson’s theory [9,23], affordances refer to the opportunities for action that an environment offers an individual, based on their abilities and perceptions. In early childhood development, affordances are crucial as they help design OPLEs that naturally encourage children to engage in activities that promote their social, emotional, cognitive, and motor development.

### 1.2. Design Domains for below-3 OPLEs

Different design domains have been identified from studies in the literature. According to several research studies, an intentional design implementation with these domains can improve the nature-based outdoor play and learning environments of below-3 children in a care facility [24]. These design domains are as follows:

- Sensory Play [25];
- Construction and Manipulation [26];
- Art, Language, and Literature [27];
- Physical Activity and Health [28];
- Rest and Relaxation [29].

#### 1.2.1. Sensory Play Opportunities

Sensory play is a key element in how infants and toddlers develop their cognitive, physical, and social–emotional domains [30]. Even from birth, babies begin to connect sensory experiences internally with movements of their extremities to explore their surroundings [31]. Young children learn by repetition, and by repeating these sensory experiences, they explore the ever-increasing movements and motions they can make [32]. Additionally, babies also use their senses to learn about people and things, connecting what they learn from their repeated internal experiences to external concepts and beings [33].

Through sensory play, these young children can explore and learn about their environment using their senses, building their neural connections, and supporting boosted brain development [34]. A typical example of sensory play with textures is playing with sand and water [35]. Infants and toddlers enhance their hand–eye coordination and fine motor skills by engaging in independent play with either of these [36]. When combined, sand and water play create an opportunity for an even more neurologically stimulating avenue of play and learning [37]. Sensory play with different smells and tastes can also help to build vocabulary and support language development [38]. An example of this kind of play could be a garden with sensory plants as well as fruits and vegetables. Overall, sensory play is a valuable tool that can be utilized to support a more holistic neurological development for infants and toddlers and is an essential aspect of high-quality early childhood education and care [14].

#### 1.2.2. Construction and Manipulation

A well-designed OPLE accommodates opportunities for manipulative play, which is critical for children’s perceptual–motor abilities and fundamental movement skill development [11]. Constructive and manipulative play greatly aids early childhood development, which helps children build skills for future learning and development [39]. Constructive play involves creating structures using materials, while manipulative play includes arranging/manipulating items to achieve specific results [40]. These activities benefit children’s social–emotional, cognitive, and motor development [15].

Constructive and manipulative play (e.g., building towers with blocks) promotes social and emotional development in infants and toddlers. These activities involve group play, allowing children to cooperate, take turns, and develop empathy [41]. Infants can substitute one thing for another by 18 months old. Constructive play like moving logs or sand and water play helps children understand space and objects [42]. By 36 months, children can estimate how objects fit and move in space and understand size and spatial terms [15]. Infants learn about their surroundings through touching and manipulating objects, developing a sense of agency. This also aids in developing fine motor skills that are essential for activities such as eating and drawing [36]. Infants as young as seven to nine months use their eyes to guide their hands when reaching for objects [43]. Lifting and squeezing objects strengthens muscles and improves motor development [44].

#### 1.2.3. Art, Language, and Literature

Art, language, and learning are all essential for healthy cognitive development in infants and toddlers as they are interconnected and significantly impact language, cognitive, social–emotional, perceptual, and motor growth [45]. Art is influential in self-expression and creativity in early childhood development by allowing infants and toddlers to explore their environment, express themselves, and develop their fine motor skills [46]. Infants and toddlers can learn about colors, shapes, textures, and patterns through art [27,47].

Language development begins in infancy when children learn to recognize the sounds and rhythms of language, which is essential for parents and caregivers to provide them with rich language experiences [48]. At eight months, infants babble back and forth with parents and caregivers [49], stop crying upon seeing someone approach to carry them, shake toys to hear the noise, and then shake again. At 18 months, they wave to parents and caregivers to gain attention [50] and squeeze toys in different directions to hear the sounds they make. At 36 months, they call out for attention while playing with their toys [51], communicate with a peer while playing, compete with complex puzzles, and show an ability to paint.

#### 1.2.4. Physical Activity and Health

Physical activity is essential for the healthy growth of infants and toddlers and is linked to their physical, psychosocial, and cognitive well-being [52]. Gross motor skills are foundational for movement and physical activity, making them critical for development [15]. Motor development is a form of behavioral development that requires infants to adapt their movements to their changing bodies and environments. Infants learn to adjust their crawling or walking based on surface qualities such as rigidity, slipperiness, or slope [44]. As children grow, they undergo physical changes that affect their motor development, necessitating freedom and opportunity for various movements [21].

Early physical activity offers numerous health benefits, including improved cardiovascular health, bone structure, and cognitive development [53]. By ages 1 and 2, children typically reach milestones like walking and throwing a ball, transitioning from independent to interactive play, which is crucial for their overall growth [54]. While children under three do not fully grasp sharing, they are more active when with peers and benefit from outdoor play that stimulates curiosity and creativity [6,55]. By increasing blood flow, oxygen, and nutrient delivery, physical activity promotes brain cell growth and cognitive function, thereby enhancing brain function [56].

#### 1.2.5. Rest and Relaxation

Rest and relaxation are crucial for the healthy development of infants and toddlers, providing essential downtime for their physical and mental growth. According to Moll et al. [57], creating spaces for rest and relaxation in outdoor play and learning environments (OPLEs) supports children’s ability to recover from physical activity and process new information. During these restful periods, the brain consolidates memories and strengthens neural connections, which is vital for cognitive development [58].

Adequate rest improves mood and behavior, making children more receptive to learning and social interactions [59]. Establishing quiet, comfortable areas in OPLEs, such as shaded spots with soft seating, encourages children to take breaks and engage in restful activities like looking at books or listening to calming music [60]. These spaces can help reduce stress and overstimulation, creating a balanced environment where children feel secure and calm [61]. Restful areas also promote the development of self-regulation skills. Infants and toddlers learn to recognize when they need a break and how to soothe themselves, which is critical for their emotional development [62]. In summary, incorporating rest and relaxation areas into the design of OPLEs supports the overall well-being of infants and toddlers by facilitating cognitive development, improving mood and behavior, and promoting self-regulation skills.

## 2. Materials and Methods

This study comprises three significant phases. Initially, the paper started with a narrative review of the existing literature. This initial review indicated that there was a lack of research focused on the outdoor play and learning experiences of below-3 children. Based on the principles of Greenhalgh, Thorne, and Malterud (2018) [63], a narrative review would be the most appropriate strategy since it provides an ‘interpretive and discursive synthesis of current material’ that can bring new insights and generate new concerns [63,64]. It may or may not specify its reference body of research using systematic search methods and inclusion/exclusion criteria, but its major focus is on the key focus of induction and interpretation in connection to the defined sample, with the goal of enhancing theoretical understanding [63]. However, narrative and methodical approaches are not mutually exclusive; a review can benefit from the strengths of both [64]. Due to the nature of our work, and the review’s connection to design applications, we considered the narrative review approach in this paper.

### 2.1. Narrative Literature Review

Narrative overviews, also called unsystematic narrative reviews, are broad syntheses of previously published literature, typically summarizing articles without employing systematic methods [65]. Narrative overviews can provoke thought, encourage discussion, and challenge existing ideas, making them useful for continuing education and professional development. While they may lack systematic methods, their role in offering a broad, accessible summary of the literature remains crucial, especially for introducing complex topics to a wider audience [65]. This study proposes the necessity of more inclusive narratives emphasizing the outdoors as a suitable environment for infants and toddlers. This generates pedagogical inquiries regarding how to create an outdoor setting for children under the age of three that can facilitate a wider range of experiences and behaviors, as well as the potential role of the educator when interacting with young children in such environments.

This study extracts the literature discussing how to improve the quality of nature-based OPLEs for children below three. This initial phase focuses on identifying design domains that connect with the developmental domains of these young children. Based on the findings from the literature studies, the second phase developed a comprehensive list of twenty outdoor play and learning settings or zones. These zones are associated with five design domains and four developmental domains, providing a framework for assessing the quality of outdoor learning environments for children below three. In the final phase, the study surveyed an existing childcare outdoor environment for children under three. Based on the twenty zones identified in Phase 2, a redesign was proposed to enhance the quality of the childcare outdoor environment.

### 2.2. Development of a List of Twenty Outdoor Play and Learning Settings/Zones

Our narrative literature review identifies twenty outdoor play and learning settings/zones. These settings are associated with five design domains and four developmental domains. We conducted a preliminary search of the literature to identify sources for OPLEs for below-three children using the Texas Tech University Libraries and Google Scholar search engines. Our search was focused on materials relevant to outdoor play and learning environments for infants and toddlers below the age of three. Our review of this body of literature yielded several relevant materials that we then synthesized to create our design domains and associated zones. Intentional design implementation of these zones can improve the nature-based outdoor play and learning environments of children below three in a care facility. The five design domains and identified associated zones are shown below in Table 1. Table 2, Table 3, Table 4, Table 5, Table 6, Table 7, Table 8, Table 9, Table 10, Table 11, Table 12, Table 13, Table 14, Table 15, Table 16, Table 17, Table 18, Table 19, Table 20 and Table 21 correspond to each of our associated zones and illustrate our synthesis of the literature that supports each zone.

### 2.3. Development of Design Guidelines and Assessment Criteria Based on Narrative Review of Literature

This research’s twenty identified zones aim to bridge the gap between current childcare facility designs and the developmental needs of infants and toddlers, offering a comprehensive approach to creating enriching, nature-based play environments. Each zone promotes diverse opportunities for sensory, motor, cognitive, and social–emotional growth. Our narrative literature review focused on below-three children, allowing us to create guidelines appropriate to this age group. Thus, the synthesis of the literature and creation of the design domains and associated zones reflect standards that are appropriate for below-three children. By developing age-appropriate design guidelines and assessment criteria, this study provides a structured framework to improve early childhood education and care environments, fostering holistic development and well-being in young children.

#### 2.3.1. Zone 1: Sand, Mud, and Earth Play

Sand, mud, and earth play are opportunities for children to explore new textures, use their hands to build new and impermanent structures, dig and discover what lies beneath the surface, and test their surroundings in a safe way that allows them to feel in control [55]. Sand’s manipulability fosters infinite play sequences that incorporate imaginative, social, and constructive play, thereby supporting informal STEAM learning [66]. An incredible and different play opportunity is created by adding water to earth play to make mud. Mud play is inherently open-ended, and a learning experience disguised as play. Specific studies (Table 2) indicate that children twelve months and older are advised to engage in sand play.

**Table 2:** Design guidelines for sand, mud, and earth play.

Design Guidelines	Literature Source
Position sand, mud, and earth play areas in a corner or adjacent to a fence, away from active areas. Refrain from situating them adjacent to a concrete pathway, which may result in slips and falls. The size of a sand play area can range from 40 to 300 square feet, depending on the age of the children and the available space. The optimal sand depth for toddlers is between 12 and 18 inches, enabling them to dig profoundly without accidentally reaching the bottom. Sand, mud, and earth play areas should be covered with materials that keep wildlife out of the pit and allow natural elements such as wind and sunlight to disinfect the used sand. Adding log slices, huge pebbles, and hooks for shovels, pails, and other items to the sand area enhances gross and fine motor development chances.	[7,55,66,67]

#### 2.3.2. Zone 2: Water Play

Gross (2012) [68] observed that water play can be the foundation upon which little future scientists discover scientific concepts of biology, chemistry, mathematics, and physics. Much like sand, water is a multisensory experience [55] that has an amazing open-ended play aspect that is magnetic for children and can be combined with other loose parts of all varieties as toddlers explore the cause and effect of mixing, splashing, bubble-making, and playing. Water exhibits a variety of intriguing properties when it is in motion. It is characterized by a highly sensory experience, the capacity to capture and reflect light, the ability to move in a variety of ways, and the remarkable capacity to induce movement in other objects [69].

**Table 3:** Design guidelines for water play.

Design Guidelines	Literature Source
As toddlers are more at risk of drowning than older age groups, safe designs for water play include hands-on water play tables, dry creek beds to allow the flow of water to be observed without dangerous pooling depth, and misters and sprayers to engage active play running through various water activities. Surfaces near water play areas should be non-skid and well-drained. It is not recommended to use harvested rainfall or standing outdoor water. Provide shade for water play and learning areas, particularly in the summer, to protect children from UV rays.	[7,55,68,69,70,71]

#### 2.3.3. Zone 3: Digging

Children create mud when they combine soil and water, which opens up a vast array of possibilities. Soil is a beautiful and intriguing substance, crucial for enriching children’s experiences. Therefore, it is recommended that digging be made available in conjunction with gardening [69]. A digging area provides toddlers with a natural environment to explore the world and develop interests, creativity, and problem-solving skills. Playing together in such areas enhances their social and emotional skills [55]. Activities like digging, burying, making mud, and creating streams teach them earth science [5]. Sandbox digging develops kinesthetic skills like balance, lifting, purposeful movement, and motor planning while also engaging their sense of touch [72].

**Table 4:** Design guidelines for digging areas.

Design Guidelines	Literature Source
To define a digging area, there must be evidence that it is intentional, which means there is enough soil/mud/dirt to dig in, scoop, and pour, and the dirt is not too tightly packed to allow for these activities. In addition, children should be given materials or equipment to use, such as shovels. Hand cleaning is necessary following digging activities. Sandboxes for digging must be properly drained, covered when unused, and maintained clean.	[5,55,72,73]

#### 2.3.4. Zone 4: Sensory Pathway

While pathways primarily serve as circulation routes, they can also provide sensory experiences [5]. Sensory pathways are crucial for the health, well-being, and development of children under three. Varying materials, textures, colors, and patterns offer motor skill challenges and sensory exploration opportunities [5,55]. These pathways can be designed to stimulate spatial relationships through different ground surfaces and plant placements, encouraging movements like up/down, in/out, and around/through [55]. As babies develop crawling and walking abilities or are wheeled to outdoor areas, diverse terrains and pathways engage them with the natural environment [74].

**Table 5:** Design guidelines for sensory pathways.

Design Guidelines	Literature Source
Design criteria for sensory pathways include the following: Changes in grade, surface, width, borders, overhead shading, perimeter rails, landscape, texture, light, color, and fragrance. Varying railing of different materials, such as wood or ropes. Interesting objects such as boulders, shrubs, activity panels, or sensory sculptures. Child-scaled benches should be included for places to rest or to allow children to engage in a lengthy sensory experience.	[5,55,74]

#### 2.3.5. Zone 5: Sensory Garden

Sensory gardens support the health, well-being, and development of children under three by fostering discovery, exploration, and interaction with a variety of plants and natural materials [2]. Diverse spaces with abundant plants encourage toddlers to explore through their senses [55]. As children process sensory information, their brains form networks essential for lifelong learning [74]. Additionally, plants stimulate the senses and enhance play settings with shade, color, fragrance, texture, and enclosure, adding substantial play value [55]. Diverse vegetation, trees, and shrubs can be employed as educational instruments to teach children about the environment and seasonal changes or as a den [75].

**Table 6:** Design guidelines for sensory gardens.

Design Guidelines	Literature Source
Design criteria for sensory gardens include the following: Objects that are rough, smooth, light, heavy, soft, hard, shiny, bright, dull, dark, large, small, natural, synthetic, thin, thick, etc. Items that make sounds or can be struck, plucked, etc., to make sound, as well as natural sounds or support for natural sounds that can be listened to and imitated. Items that have a variety of smells. Things to look at that are both high and low. For infants, creating an intimate multisensory environment and providing ample opportunities for ground-level exploration of various objects and surfaces is essential.	[5,55,74]

#### 2.3.6. Zone 6: Music

Curtis and Carter [74] mentioned that children might feel powerfully significant in their tiny bodies by making loud noises. Youngsters frequently utilize sounds instead of words when they play. It is important to allow kids to drum and provide opportunities to bang loudly outside to foster their curiosity [74]. Petruta-Maria (2015) [76] demonstrated that music is therapeutic for children with special needs.

**Table 7:** Design guidelines for music.

Design Guidelines	Literature Source
Use of different materials that create different sounds to teach children about the properties of materials. Adding a variety of rattles and other noisemakers in the sensory nook. Providing places for auditory development and using wind chimes, gongs, and other materials that can create sound with natural phenomena or have properties to produce sound when thumped. Hang wind chimes or digital devices to create visual and acoustic interest.	[2,5,7,74,76,77]

#### 2.3.7. Zone 7: Natural Construction

Construction play is significantly expanded by including it as a significant component of outdoor provision, as children will have access to an extensive amount of space, freedom, and stimuli. This allows children to engage in a plethora of activities that are pertinent and meaningful to them, thereby enabling children to further explore these big questions [69]. Natural construction inspires STEAM (science, technology, engineering, art, and math) activities, fostering imagination and development through open-ended play [55]. Daly and Beloglovsky (2014) [78] demonstrate how outdoor play with materials at an early age awakens creativity as children gather information, think, and experiment. Greenman and Greenman (2005) [5] emphasize the importance of spaces for constructive play, noting that children build shelters and structures outdoors.

**Table 8:** Design guidelines for natural construction.

Design Guidelines	Literature Source
It is recommended that the environment is consistently supplied with readily available natural loose materials, preferably stored on racks. Choose a shaded place with trees, logs, fences, walls, or posts to offer support for children to build on.	[5,55,69,78,79]

#### 2.3.8. Zone 8: Loose Parts Play

Nicholson (1971) [80] described loose parts as open-ended objects and materials that children can manipulate in various ways. Anggard (2011) [81] noted that loose parts allow children to play based on their desires, unlike fixed materials. These materials range from small objects to large sets of manufactured and natural items, offering multiple affordances with no specific directions and enabling creative action [55]. Flannigan and Dietze (2017) [82] highlighted that loose parts promote play opportunities, social interaction, and risk-taking. Providing loose parts early on extends play and learning possibilities, reducing negative attitudes toward play’s developmental value [83].

**Table 9:** Design guidelines for loose parts play.

Design Guidelines	Literature Source
Locate loose activity areas near natural features that provide ample play and learning opportunities, such as trees, shrubs, long grasses, and pickable flowers. Provide shade for children during extended play sessions with trees, pergolas, umbrellas, or shade sails. Loose parts in areas for infants and toddlers should be monitored closely to rule out choking hazards. All unsafe parts must be inspected, and hazardous or defective objects must be removed immediately.	[55,78,80,82,83,84]

#### 2.3.9. Zone 9: Wildlife Garden

The inclusion of wildlife gardens can also support the health, well-being, and development of children under the age of 3. Wildlife gardens can encourage children to gain empathy and respect for various life forms, increase their understanding of ecology and life processes in the natural world, and provide living resources for nature education [55]. Additionally, various animals, such as caterpillars, butterflies, worms, ladybugs, and snails, can stimulate exploration and discovery and enhance outdoor education [85]. It is advisable to have plants growing throughout the outdoor area rather than constrained to a single horticultural or wildlife area, as children benefit from this element being an integral part of the entire space [69].

**Table 10:** Design guidelines for wildlife gardens.

Design Guidelines	Literature Source
Opportunities to observe insects, birds, and small creatures. Diverse plants with varying heights, flowering periods, and growth types for animal habitat. Vertical diversity ensures year-round food and shelter for different species. Bird and butterfly feeders and plants like butterfly bushes let kids watch wildlife and ensure hands-on involvement with nature. For predator protection, bird feeders should be away from classroom windows and within 10–15 feet of shrubby vegetation. Furthermore, native plants should be considered as they are the best choice for supporting local pollinating insect populations.	[7,55,69,85]

#### 2.3.10. Zone 10: Art

Koster and Linton (2014) [86] found that teaching the arts to young children fosters development in understanding cause and effect, pattern recognition, organization, and rational thinking while also promoting community building beyond racial boundaries. Schwarz and Luckenbill (2012) [87] noted that involving infants and toddlers in art activities enhances cognitive development through exposure to various materials and their tactile qualities. Curtis and Carter (2014) [74] emphasize the use of warm colors, textures, and artwork to enhance emotional and physical relaxation. Rubin (2005) [88] highlights art as a crucial non-verbal communication tool and therapeutic method for children with disabilities. There are numerous factors that motivate children to draw outdoors, particularly those that are part of the natural world. This is the point at which a well-stocked mobile trolley or toolset becomes especially crucial in order to prevent the occurrence of losing the moment [69].

**Table 11:** Design guidelines for art.

Design Guidelines	Literature Source
The use of non-toxic elements as a paint material is critical since infants and toddlers tend to put things in their mouths. Washable, non-toxic materials must be used, and small parts must not be contained.	[69,74,86,87]

#### 2.3.11. Zone 11: Outdoor Reading and Language Play

Moore (2014) [55] discusses that children aged 0–18 months love listening and try to imitate sounds. Greenman (2005) [5] emphasizes providing places to have conversations with children and that “the play environment should be developed as a wonderful, interesting place that continually captures a child’s attention and is laid out to ensure individual and group experiences”. A storytelling nook or outdoor reading area provides an area for such group activities. According to Curtis and Carter (2014) [74], children symbolically understand their surroundings. Storytelling is the most effective way of learning language by providing an opportunity to learn a range of vocabulary and sentence structure [89].

**Table 12:** Design guidelines for outdoor reading and language play.

Design Guidelines	Literature Source
Use natural elements to attract birds and insects into outdoor learning environmental design to aid children with language literacy. Use different types of signage to create a certain mode of communication throughout the site. Use tools and materials with images, as well as representative symbols, to aid language and reading skills.	[55,74,89]

#### 2.3.12. Zone 12: Pretend and Performance Area

Pretend and performance play is an essential activity in child development. Play structures or materials that contain ambiguity provide opportunities for children’s imagination to take over and to pretend play [5]. Performance can stimulate self-expression, encourage teamwork, foster a sense of community, and create a “culture of place” [55]. The outdoors is a natural site for performance and pretending. It creates an expression that enables children to discover their relationship to life and beauty, as provided by play materials [5]. Morrissey (2014) [90] suggests that when children are surrounded and supported by an appropriate mix of reality and abstract play materials from 8–9 months of age, their capacity to participate in such activity is triggered.

**Table 13:** Design guidelines for pretend and performance areas.

Design Guidelines	Literature Source
Create gathering spaces such as a deck, circle, or small amphitheater that accommodates groups. Consider the orientation of sun and shade in creating pretend and performance play areas. If the space needs to accommodate large audiences, locate it for convenient and direct access.	[55,90]

#### 2.3.13. Zone 13: Pathways for Play and Movement

The primary focus of young children is the development of a perception of their body and its position in space as it relates to gravity and other objects [69]. Moore (2014) [55] emphasizes the critical role of pathways for creating “movement sequences” for toddlerhood, an age range when spatial exploration expands dramatically. Greenman (2005) [5] points out the need for “Places for Running” in early childhood outdoor spaces, noting that toddlers and other unsteady walkers need “smooth, flat surfaces” for pathways and tracks designed to promote movement. Greenman (2005) [5] further emphasizes the importance of pathways for providing affordances to children to journey, haul, race, ferry, and caravan between two points. Pathways are repeatedly mentioned as a vital landscape component for play and learning environments for preschool-aged (3–5 years old) children.

**Table 14:** Design guidelines for pathways for play and movement.

Design Guidelines	Literature Source
Use a looping form of the pathway that connects to significant play and learning activities. Make sure the pathway is ADA-accessible. Curve the pathways to create a sense of discovery and exploration. Consider secondary and tertiary pathways to increase accessibility and alternative connections. Consider using transport such as wagons, wheelbarrows, strollers, hauling, etc. Engage children by using changes in grade, surface material, width, borders, shading, rails, landscaping, benches, and activity panels.	[5,55,69,74]

#### 2.3.14. Zone 14: Play Structures

Physical activities in outdoor spaces are the most anticipated affordance in a designed playscape. Moore (2014) [55] notes that physical movement for children below three is one of the most significant modes of learning. Children learn about themselves and their abilities by practicing and expanding their physical skills. While installing any play structure, Greenman (2005) [5] emphasizes prioritizing children’s nature to continuously explore new challenges. He suggested that play structures should provide the chance to change and re-create to diversify opportunities for play engagement. Harrison (1990) [2] states that creating stimulating and challenging learning experiences for children below three fosters their independence and promotes their freedom to make choices.

**Table 15:** Design guidelines for play structures.

Design Guidelines	Literature Source
Use play structures made from natural materials such as logs and rocks. Consider engaging local artisans and using native materials.	[2,5,55,74]

#### 2.3.15. Zone 15: Landforms and Topography

When designing an outdoor space for children, it is crucial to consider a selection of vibrant plants, materials, ground covers, and landscape features, such as hills and puddles. Moore (2014) [55] highlights landforms as the fundamental element of the terrestrial environment. He suggests that landforms can inspire play and facilitate learning about nature by observing various natural phenomena, such as light and shadow patterns on the landform and water drainage. Landforms provide opportunities for excellent physical activity; alterations in elevation facilitate climbing, rolling, leaping, and sliding. Landforms can also be modified to offer a sense of enclosure and open, high, and low spatial experiences [75].

**Table 16:** Design guidelines for landforms and topography.

Design Guidelines	Literature Source
Use landforms to create slides, tunnels, and caves. Design landforms to encourage the development of children’s motor, sensory, and orientation skills. Use landforms to create visual barriers and a sense of enclosure. Safe crawling areas include grass, vinyl, or wood composite decking and sturdy railings at 14-16 inches for newborns to stand on. A safe play area should include a non-metal slide with a gentle slope, short tunnels, and peek-a-boo areas, and seating at various levels.	[5,7,74,75]

#### 2.3.16. Zone 16: Multipurpose Lawn

Moore (2014) [55] underscored the continuous green lawn as the most desired feature of a learning playscape, especially for toddlers who are learning to walk. However, it is also essential to consider scale, maintenance, and connectivity to pathways and adjacent functional settings. Lawns can support multiple affordances, like spaces for informal games, group activities (assembled by caregivers), and performance spaces. Herrington and Studtmann (1998) [91] observed that children react differently on a grassy lawn than in a playground equipped with various play structures. They found that children’s imaginative abilities dominate physical abilities in a vegetative outdoor playground.

**Table 17:** Design guidelines for multipurpose lawns.

Design Guidelines	Literature Source
Consider whether soil conditions or irrigation support a lawn. Consider the overall space size and whether including a lawn is appropriate. Consider the users and uses of the lawn space.	[55,74,91]

#### 2.3.17. Zone 17: Fruit and Vegetable Garden

Fruit and vegetable gardens are an important aspect of the playscape for toddlers. The act of planting, cultivating, and consuming fruits and vegetables provides a wealth of knowledge and enjoyment. For children, the produce they grow is of interest to them, and it is something they will want to consume [69]. From an early age, fruit and vegetable gardens teach toddlers about responsibility and stewardship of the earth, provide opportunities to work alongside each other, develop a sense of community, and afford a place to take safe risks and learn from teachers [26]. Additionally, fruit and vegetable gardens deliver opportunities for toddlers to develop a respect for living things around them, including small insects and wildlife that will be attracted to the garden [92].

**Table 18:** Design guidelines for fruit and vegetable gardens.

Design Guidelines	Literature Source
Consider the heights and capabilities of the users and provide a variety of planting heights. Include a design that is ADA-accessible. Provide ample space for circulation. Include opportunities for professionals and educators to manage the garden and teach children.	[55,69,74,92]

#### 2.3.18. Zone 18: Resting and Nap Area

Resting and sleeping outside create memorable experiences for children [5]. Private resting areas allow children to be alone for rest, quiet, or to deal with some of their feelings when needed [93]. A well-designed resting area helps infants and toddlers calm down and enjoy a comfortable, peaceful environment. Children discover that one section of the room is appropriate for jumping, running, climbing, and falling, while the peaceful rest area is not. Making everything safe and healthy is the first step in ensuring a comfortable environment [93].

**Table 19:** Design guidelines for resting and nap areas.

Design Guidelines	Literature Source
Divide spaces to create a separate resting area apart from loud spaces. Use soft, natural colors and provide soft furnishings. A porch swing or hammock in a shaded place is perfect for feeding and raising infants while also providing comfort to caregivers.	[7,74,93]

#### 2.3.19. Zone 19: Utility Zone

The utility zone can serve as a program base for the outdoor learning environment and can include storage for tools, equipment, and materials [55]. Having adequate storage can significantly impact the viability of hands-on programming [55]. Additionally, children may be inspired to tidy up after playing if the storage area is clearly defined, labeled, and strategically placed [94]. The provision of enough storage space helps to reduce the clutter that hinders regular play activities. Furthermore, an orderly environment where toys and materials are stored in the same place each day, predictable routines, and children’s cots or mats in the exact location are helpful for children to develop long-term memory and awareness of location and space [95].

**Table 20:** Design guidelines for utility zones.

Design Guidelines	Literature Source
Consider the size and location of storage to increase the viability of hands-on programming. Ensure the space is easily accessible and clearly signposted. As a program base, consider this space as a social anchor, a central space for communication and emergency first aid. Consider including shaded areas for eating and lounging, as well as easy access to diapering and hand-washing stations.	[7,55,74,95]

#### 2.3.20. Zone 20: Natural Healing and Relaxation

Outdoor play and learning environments can provide unique natural healing and relaxation opportunities. Natural experiences, such as breezes, sunlight, grass, sand, the smell of trees, and light on water, soothe and refresh people [93]. Connection to nature and plantings, natural materials, nature sounds, and the presence of water provide practical therapeutic benefits [96]. Vegetated circles and nooks can provide secluded spaces for children to escape, relax, and interact with nature [55].

**Table 21:** Design guidelines for natural healing and relaxation.

Design Guidelines	Literature Source
Incorporate natural patterns and variations to provide gentle changes in stimulation and encourage relaxed alertness and feelings of comfort. Include native plants that may boost the human immune system in your design. Site these spaces in a quiet location.	[55,74,93,96]

## 3. Environmental Assessment Review

In the concluding phase of the study, a comprehensive survey was conducted to evaluate the existing outdoor environment at a childcare facility catering to below-three children. The site assessed in this study is the Texas Tech University Early Head Start located in east Lubbock, Texas. The Early Head Start program is a child development and family support program for children from 0 to 3 years of age. Based on the groundwork laid in Phase 2, where twenty distinct zones were identified, the survey aimed to ascertain the functional and recreational adequacy of the areas. A survey was created and administered using Qualtrics. The survey measured the presence of play and learning opportunities in the five design domains and each of the twenty associated zones. The measurement was based on a rating scale of 0 to 5. The survey aimed to ascertain the impact of designing an outdoor play and learning environment for below-three children following the guidelines from our narrative literature review outlined above in Part 2. The survey was conducted prior to the design intervention to evaluate the site’s existing settings and to better understand its play and learning needs. The results from this survey, along with the guidelines created from our narrative literature review, guided our redesign of the site. The survey was then conducted again based on the site redesign so that we could compare before and after assessments to measure the impact our guidelines have on outdoor play and learning environments for below-three children. The results of our before-and-after analysis are presented below.

### 3.1. Proposed Design for Nature-Based OPLEs for Below-Three Children

**Figure 1:**
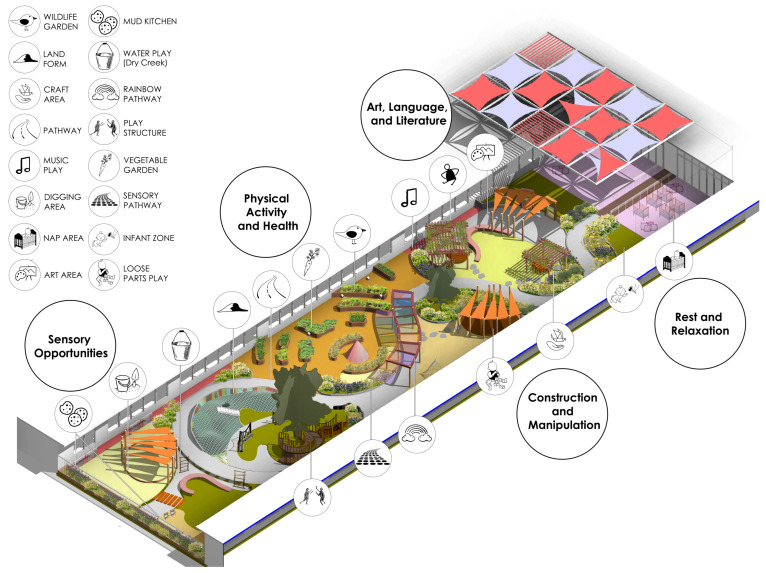
Proposed design for nature-based OPLEs for below-three children.

**Figure 2:**
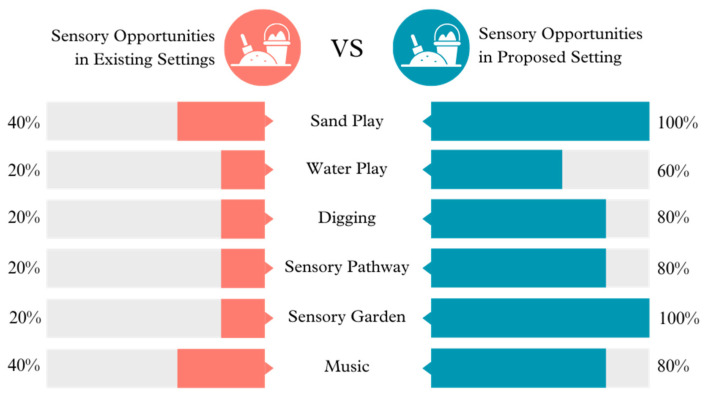
Sensory opportunities in existing vs. proposed settings.

**Figure 3:**
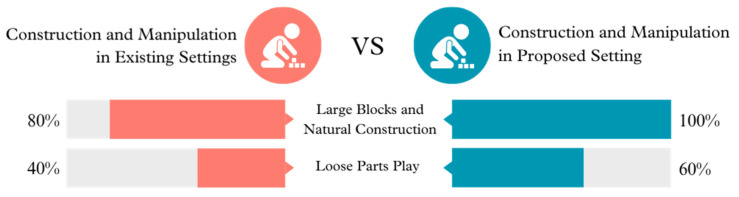
Construction and manipulation opportunities in existing vs. proposed settings.

**Figure 4:**
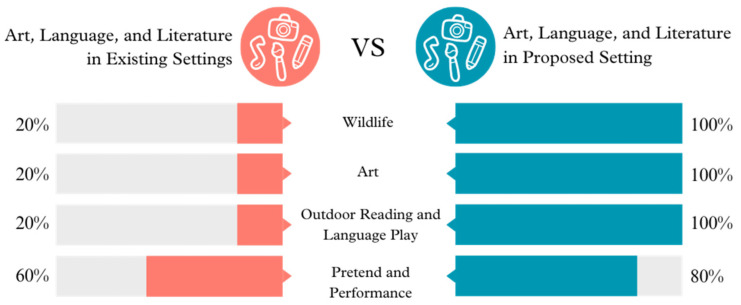
Art, language, and literature opportunities in existing vs. proposed settings.

**Figure 5:**
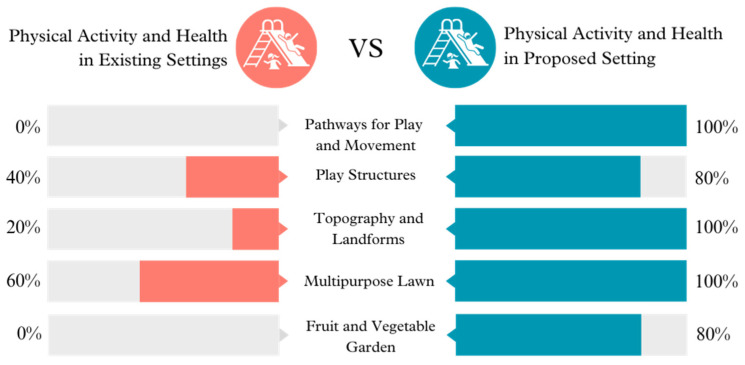
Physical activity and health opportunities in existing vs. proposed settings.

**Figure 6:**
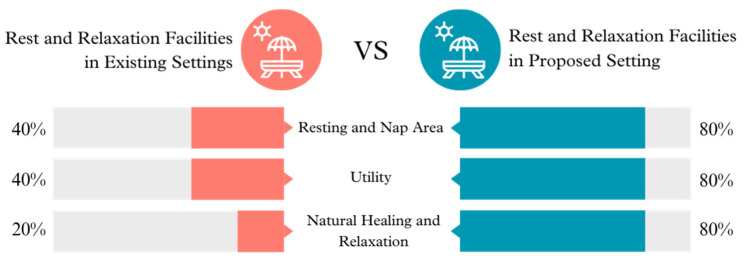
Rest and relaxation facilities in existing vs. proposed settings.

By focusing on the developmental domains of young children cited in the literature, the design creates (Figure 1) interactive, challenging, and explorative spaces that support language development, cognitive development, motor and perceptual skills development, and social and emotional development. The design integrates research, data, and feedback for engaging nature play and learning for young children. Our survey results yield firsthand data pertaining to the below-three OPLE guidelines that were developed from our narrative literature review. The feedback from the survey directly informed our design intervention, as described in more detail below. Figure 2, Figure 3, Figure 4, Figure 5 and Figure 6 show the survey results in the existing and proposed settings and further illustrate how the proposed design intervention based on the narrative literature review improved the overall site functioning as an OPLE. Thus, the narrative literature review, data, and feedback are integrated into the proposed design.

The proposed design is inclusive for all children under three years of age. The design allows children to have unique experiences each day and provides many learning affordances. The ADA-accessible looped pathway encourages all children to explore the entire play area, and secondary pathways offer opportunities for additional exploration and adventure. The playground design considers the distinct age groups within the 6-month to 3-year range—infants and toddlers—to prevent conflicts. The infant zone is placed near the entrance to keep it calm and accessible for caregivers. In contrast, the more active toddler zone is positioned farther outside to minimize noise and activity at the entrance. The semi-shaded infant zone allows easy caregiver assistance with changing and feeding, creating a smooth transition between indoor and outdoor spaces. Special attention was given to the planting palette, which provides appropriate natural play materials for young children. The environment creates engaging opportunities for parallel play, nature learning, small group interaction, and relevant challenges that support child development. 

### 3.2. Comparison of Pre- and Post-Design Assessment Scores of the Selected Childcare OPLE

The findings from the survey represent a comparative analysis of opportunities and facilities in existing versus proposed settings of the selected childcare outdoor environment. The comparative analysis is grouped into the five design domains: Sensory Opportunities, Construction and Manipulation, Art, Language, and Literature, Physical Activity and Health, and Rest and Relaxation Facilities. The existing setting survey results reflect the presence of aspects of the design domains and associated zones in the site prior to the design intervention. The proposed setting survey results reflect the presence of aspects of the design domains and associated zones with our new design implemented in the site (see Figure 1). Figure 2, Figure 3, Figure 4, Figure 5 and Figure 6 show the comparative analysis based on six landscape architecture students filling out the surveys for the existing site and the proposed site design. As discussed below, the results reflect an increase in outdoor play and learning environment affordances with the proposed site design implemented.

Sensory opportunities: The proposed environment significantly increases all sensory experiences, such as Sand Play, Water Play, Digging, Sensory Pathways, Sensory Garden, and Music. The current settings offer limited sensory experiences, indicating an untapped potential for sensory engagement. The proposed settings are significantly more comprehensive, perhaps providing more varied and immersive experiences essential for young children’s development.

The proposed setting incorporates and enhances the use of Large Blocks and Natural Construction, as well as Loose Parts Play. Engaging in these activities is crucial for the development of fine motor skills and cognitive talents, including problem-solving and creativity. The current environment appears to provide a limited range of materials and opportunities for building and manipulating objects, which may impede the children’s ability to engage in exploratory and imaginative play.

Significant enhancements are evident in the suggested configuration for Wildlife interaction, Art, Outdoor Reading and Language Play, and Pretend and Performance. These improvements indicate a purposeful attempt to incorporate educational and creative activities into the outdoor setting, promoting a comprehensive developmental environment.

The redesign prioritizes enhancing Physical Activity and Health by upgrading Pathways for Play and Movement, Play Structures, Topography and Landforms, a Multipurpose Lawn, and a Fruit and Vegetable Garden. These modifications promote physical exercise and engagement with the environment, which are crucial for maintaining physical well-being and developing a comprehensive understanding of the natural world.

The proposed configuration improves the Rest and Relaxation amenities by enhancing the Resting and Nap Area, introducing Utility amenities, and integrating Natural Healing and Relaxation places. These improvements demonstrate a recognition of the significance of rest and relaxation in children’s daily routines, with a noticeable upgrade from the current facilities, which appear simplistic and less favorable for relaxation.

The findings from this comparative analysis revealed a significant improvement in the overall assessment score, indicating that the inclusion of twenty zones in the redesigned outdoor environment substantially exceeds the quality of the original setup. This radical enhancement underscores the importance of a well-thought-out design and its profound impact on the developmental opportunities available to children in childcare settings. The study’s approach demonstrates a successful application of environmental and developmental psychology principles to create a space that not only meets the basic needs of young children but also actively contributes to their cognitive, emotional, and physical development through well-designed outdoor play and learning spaces.

## 4. Conclusions

In agreement with Da Costa et al. (2021) [97], this study supports that babies and young children can exercise their rights and learn about the world through hands-on experience without needing adult-centered explanations by having access to nature and outdoor gathering spaces. This paper sought to identify and evaluate indicators for improving nature-based outdoor play and learning environments for children below three based on relevant literature. By focusing on four key developmental domains—social–emotional, language, cognitive, and perceptual–motor—this research established five critical design domains, leading to the creation of twenty specific outdoor play and learning zones. Design guidelines for each of the zones were summarized and then applied to an existing OPLE at a childcare facility that caters to below-three children to inform a proposed redesign of the space.

Survey assessments were applied to the OPLE in its existing condition, and the proposed redesign and results indicate increased functionality and adequacy of the OPLE. The OPLE assessment shows how the identified indicators for improving OPLEs in below-three children can create spaces that support child development across recognized developmental domains and enrich their learning and play environments. The design domains and associated zones are applicable across diverse OPLE settings, and the guidelines described herein are highly adaptable to improve OPLEs. The findings underscore the value of thoughtfully designed nature-based environments in promoting holistic child development and offer adaptable guidelines for childcare settings. This research contributes valuable insights for educators, designers, and policymakers aiming to foster enriched, nature-integrated learning experiences for below-three children.

## Figures and Tables

**Table 1:** Five design domains and associated zones.

Design Domains	Associated Zones
1. Sensory Opportunities	1.1 Zone 1: Sand/Mud Play
	1.2 Zone 2: Water Play
	1.3 Zone 3: Digging
	1.4 Zone 4: Sensory Pathway
	1.5 Zone 5: Sensory Garden
	1.6 Zone 6: Music
2. Construction and Manipulation	2.1 Zone 7: Large Blocks and Natural Construction
	2.2 Zone 8: Loose Parts Play
3. Art, Language, and Literature	3.1 Zone 9: Wildlife
	3.2 Zone 10: Art
	3.3 Zone 11: Outdoor Reading and Language Play
	3.4 Zone 12: Pretend and Performance
4. Physical Activity and Health	4.1 Zone 13: Pathways for Play and Movement
	4.2 Zone 14: Play Structures
	4.3 Zone 15: Topography and Landforms
	4.4 Zone 16: Multipurpose Lawn4.5 Zone 17: Fruit and Vegetable Garden
5. Rest and Relaxation	5.1 Zone 18: Resting and Nap Area
	5.2 Zone 19: Utility
	5.3 Zone 20: Natural Healing and Relaxation
